# Supplementary material for: Zooming into Gut Dysbiosis in Parkinson’s Disease: New Insights from Functional Mapping
Source: Int J Mol Sci. 2023 Jun 5;24(11):9777. doi: 10.3390/ijms24119777 (PMC10253733; doi:10.3390/ijms24119777)
Supplement: Supplementary file 1 [file ijms-24-09777-s001.zip › ijms-2346034-supplementary.pdf]

Figure S1.

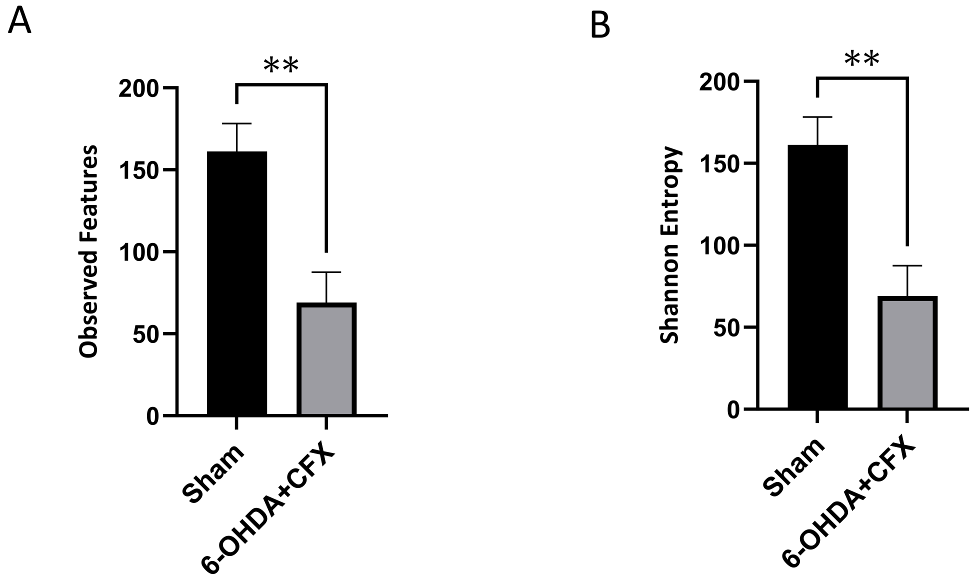

Figure S1. Gut microbiota diversity of Sham and 6-OHDA+CFX groups. A-B) Observed Features and Shannon Entropy, respectively, presented as mean ± SEM. Asterisks indicate a significant difference as obtained by the Unpaired t-test (**p < 0.01).
